# Weak Antilocalization and Quantum Oscillations of Surface States in Topologically Nontrivial DyPdBi(110)Half Heusler alloy

**DOI:** 10.1038/s41598-018-28382-1

**Published:** 2018-07-02

**Authors:** Vishal Bhardwaj, Satyendra Prakash Pal, Lajos K. Varga, Monika Tomar, Vinay Gupta, Ratnamala Chatterjee

**Affiliations:** 1 Department of Physics, Indian Institute of Technology Delhi, Hauz Khas, New Delhi 110016 India; 2 Wigner research center for Physics Hungarian Academy of Sciences, P.O.B. 49, H-1525 Budapest, Hungary; 3 Department of Physics and Astrophysics, University of Delhi, Delhi, 110007 India

## Abstract

Recently, a number of ternary half-Heusler compounds have been predicted independently by several research groups as candidates for 3D topological insulators. In this work, we report the observation of a two-dimensional (2D) weak antilocalization (WAL) effect, one of the hall-marks of topological surface states, and Shubnikov-de Hass (SdH) quantum oscillations in <110> oriented DyPdBi (DPB) thin films grown on MgO (100) substrates. The films prepared by pulsed laser deposition technique under the optimized conditions, showed a textured structure with (110) planes parallel to the (100) plane of MgO. The measured WAL effect follows the Hikami-Larkin-Nagaoka (HLN) model and the extracted values of phase coherence length (*l*_ϕ_) and α are ~420 nm and ~−0.52 respectively. The power law variation of *l*_ϕ_ (~T^−0.46^) indicates the presence of the 2D surface states in DPB film. The Dirac nature of the surface states is further confirmed by Landau-level fan diagram analysis of SdH oscillations of the magneto-transport data. This analysis shows a finite Berry phase of 0.90π ± 0.16, reasonably close to the expected π value. Sheet Carrier density, n_s_ ~ 2.56 × 10^12^ cm^−2^, calculated from the SdH oscillations (*f*_SdH_ ~ 106 T) and Hall measurements agree well with each other. These findings demonstrate that the half Heusler DPB thin films (~15–20 nm) can be used as a suitable material for investigating the novel intrinsic quantum transport properties of surface Dirac fermions.

## Introduction

The topological insulator (TI) is a new quantum state of matter where insulating bulk states coexist with topologically protected spin polarized surface (edge) states in 3D (2D) TI^1^. These surface/edge helical states are protected by time-reversal-symmetry^2,3^ induced by a strong spin-orbit coupling^4,5^. Currently the most extensively studied materials are HgTe/CdTe quantum wells and (Bi/Sb)_2_(Te/Se)_3_ based 3D TIs^5–8^.

Very recently, a number of ternary half-Heusler compounds have been theoretically predicted by several research groups to be candidates for 3D TIs^9–11^. Half-Heusler compounds form a vast group of cubic ternary intermetallic alloys that crystallize in MgAgAs-type structure with composition *XYZ* that are derived from cubic full Heusler alloys (*X*_2_*YZ)* named after their discoverer, Fritz Heusler^12^. The X and Y are transition or rare-earth elements and Z is a heavy metal. The crucial ingredient in these intermetallics is the presence of strong spin-orbit coupling (SOC) heavy metal that gives rise to an additional requirement (band inversion) to drive these systems into a topological phase. Yan *et al*.^13^ have shown that the electronic properties of Half Heusler alloys can be predicted by counting the sum of valence electrons of constituent elements. If sum is equal to 8 or 18 electrons, they show semiconductor properties just like classical semiconductors, such as GaAs. Band gap of these alloys can be tuned in wide range from 4 eV (e.g., LiMgN) down to zero(e.g., ScPtBi), by choosing X, Y, Z with varying electronegativity and lattice constant values^13^.

Rare earth (R) based RPdBi half Heusler alloys were predicted to exist in either trivial or topological state depending on their equilibrium lattice constant value in a recent work done by Nakajima *et al*.^11^. DPB (6.63 Å) was predicted to exist at the border between trivial and topological state. Also in an earlier work done by Chadov *et al*.^10^, it is theoretically predicted that one can drive YPdBi (6.625 Å, which also sits at the border line), from trivial state to topological state by applying tensile strain in its lattice constant.

In this work we report our results on <110> DPB thin films (~15–20 nm thicknesses) grown on single crystal MgO (100) substrates. We highlight the observations of WAL and SdH oscillations arising from the surface states in the magneto-transport data obtained on the DPB thin films. The WAL is sensitive only to perpendicular magnetic field component and is described well by HLN model. The estimated *l*_ϕ_ varies with the temperature T as per the power-law (*l*_ϕ_ ~ T^−0.46^), demonstrating the presence of 2D surface states. The high magnetic field magneto-transport data shows SdH oscillations and reveal the presence of 2D Fermi surface with Fermi vector (k_F_ ∼ 0.0568 Å^−1^) in the DPB thin film that survive up to 10 K. Landau level fan diagram analysis of the SdH oscillations gives a finite Berry phase of 0.90π ± 0.16 pointing out Dirac dispersion of massless Dirac fermions and the presence of topologically protected surface states.

## Results

### Structural characterizations

**Figure 1:**
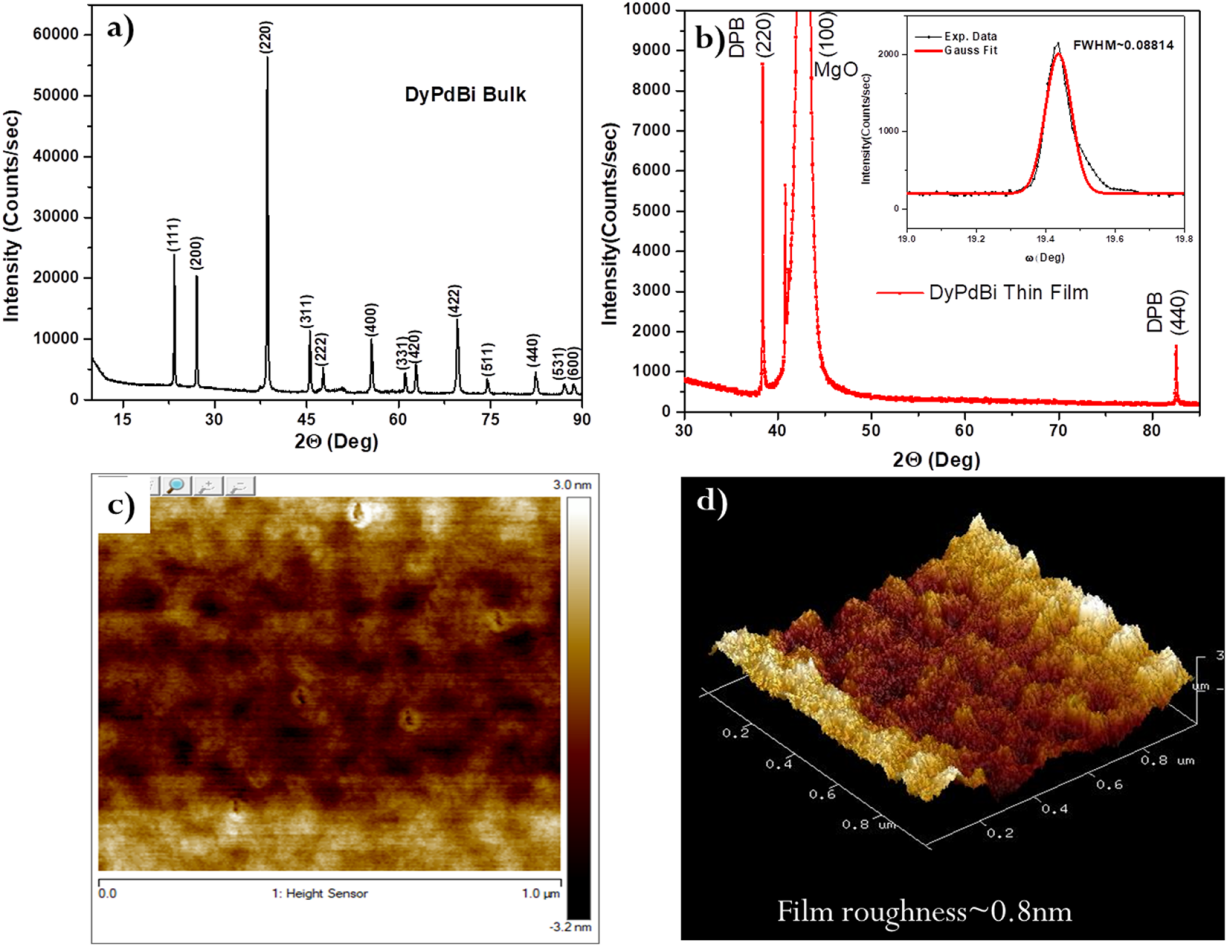
Structural characterizations of DPB. (**a**) Powder XRD pattern of bulk DPB polycrystalline sample. (**b**) Gonio-mode XRD pattern of DPB thin film with [110] oriented planes. Inset shows rocking curve of (220)-Bragg reflection with FWHM value ~0.088°. (**c**) AFM topographic image of DPB thin film with thickness ~20 nm. (**d**) Tilted 3D AFM image of same sample showing roughness ~0.8 nm.

Polycrystalline bulk sample of DPB was prepared in a specially designed RF induction melting set-up, as described in the Methods section. The low melting temperature of Bi makes it hard to maintain the stoichiometry ratio of the constituent elements. We could achieve the stoichiometric DPB compound, starting with the optimized amounts of constituent elements, in this specially designed RF induction melting furnace. Figure 1a shows a representative powder X-ray diffraction pattern of the bulk DPB sample. All peaks are well indexed to a MgAgAs-type structure with a lattice constant of (6.63 Å), which is consistent with the values obtained in the previous reports^11,14,15^. The <110> oriented DPB thin films of 15 ~ 20 nm were grown at optimized substrate temperature (500 °C) (see supplementary material Fig. S1a), on MgO(100) with Ta (5 nm) as seed layer, using pulsed laser ablation; as described in Methods section. Figure 1b shows the X-ray diffraction pattern of <110> DPB thin film sample recorded in gonio-mode. Oriented [110] growth is clearly evidenced from this figure. Rocking curve analysis of [220] peak shows small FWHM value ~0.088° and thus provides further confirmation of oriented growth. Although both DPB (C1_b,_ a ~ 6.63 Å) and MgO (B_1_, a ~ 4.21 Å) have cubic structure, along (100) cubic axis, there is a large lattice mismatch (~59%) between them. However, an architecture shown in schematic Fig. S1b (Supplementary), clearly depicts that the mismatch remains only around 0.2% for DPB (110) plane (a_110_ ~ 9.38 Å) on rotated MgO (100) plane (~9.40 Å). Thus, MgO (100) substrate is conducive for <110> oriented growth of DPB films.

Thickness (15 ~ 20 nm) of thin films was estimated using X Ray Reflectivity (see supplementary material Fig. S2). Atomic force microscopy (AFM) revealed roughness ~0.8 nm in a 20 nm thick film (Fig. 1c,d); further results in this report are obtained on the same sample. A scanning electron microscope with energy-dispersive X-ray spectrometry (SEM-EDX) was used to estimate the composition of DPB thin films. EDX (see supplementary material Figs S3, S4 and Tables S1, S2) shows that the DPB thin film have calculated average atomic % of Dy, Pd, Bi as 33.08%, 33.42% and 33.50%,respectively for different regions of sample.

### WAL effect in DPB

**Figure 2:**
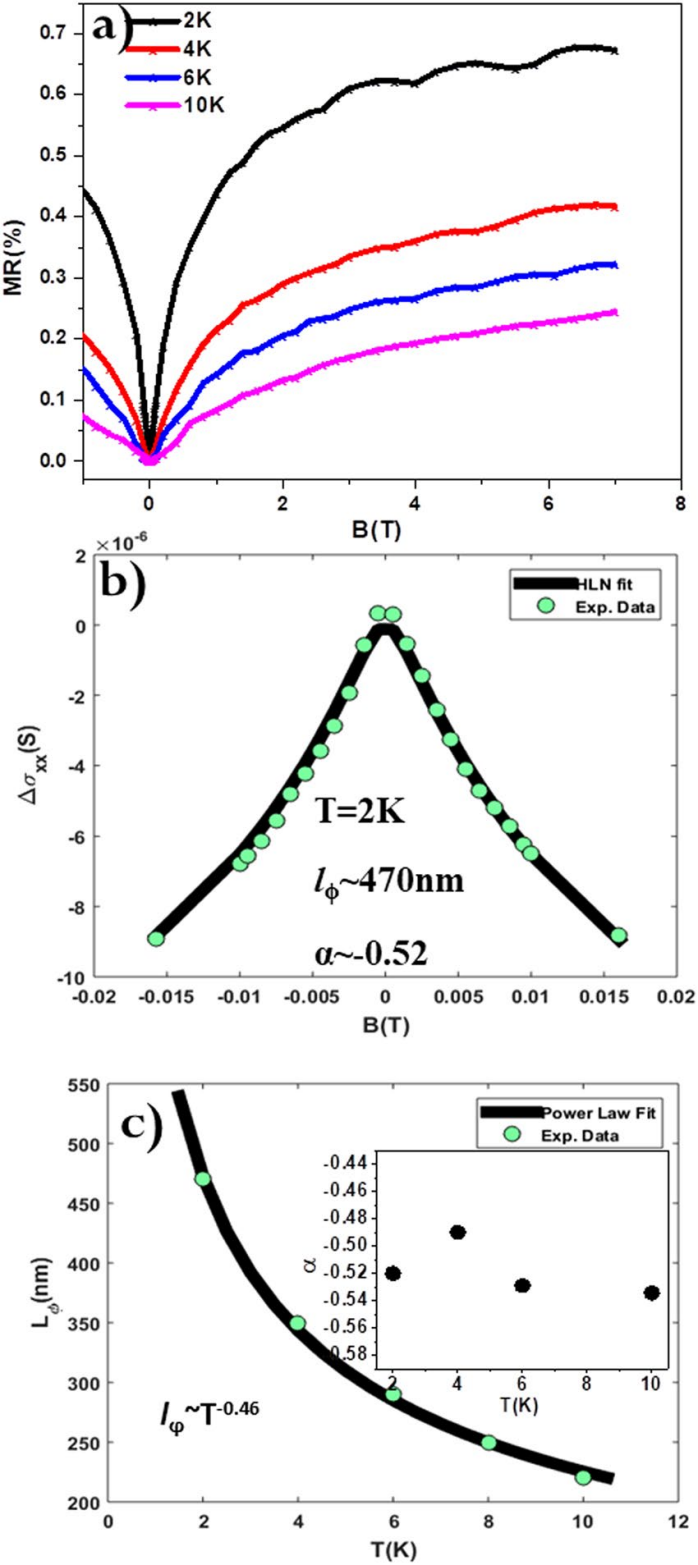
WAL behavior in DPB. (**a**) Normalized MR as a function of magnetic field H at a series of temperatures T = 2, 4, 6 and 10 K. Here MR = [ρ (H) − ρ (0)]/ρ (0)] × 100%, where ρ (H) and ρ (0) are the resistivities with and without the magnetic field H, respectively. (**b**) HLN fitting (solid black line) of 2 K data (cyan color dots) around low magnetic field region. The corresponding fitting parameters obtained are α ~ −0.52 and *l*_ϕ_ ~ 470 nm, which confirm the 2D nature of WAL. (**c**) Variation of *l*_ϕ_ with temperature (2 K ≤ T ≤ 10 K). The solid line (black) shows the power law variation of *l*_ϕ_ with temperature as *l*_ϕ_ ~ T^−0.46^, inset shows variation of prefactor α (from HLN theory) with temperature.

Figure 2a shows the magnetic-field dependence of the normalized Magneto resistance (MR) at different temperatures. The MR is defined as [*ρ*(H) − *ρ*(0)]/*ρ*(0)] × 100%, where *ρ*(H) and *ρ*(0) are the resistivities at magnetic field H and zero field, respectively. A very small MR (0.7%) was observed under a 7 T magnetic field at 2 K. A prominent cusp of MR observed in the low magnetic field region (Fig. 2a) indicates WAL effect originating from the π Berry’s phase related to the helical states of the charge carriers in the film. This observed WAL effect in the DPB thin films is a key signature of Dirac nature of topological surface states and strong SOC. This effect has been widely reported in BiSeTe^16,17^, BiSe^7^ based TIs, and recently in single crystal samples of half Heusler alloys^18–20^ based TIs. However, since our experimental conditions only permit the measurements in the condition θ = 0, one cannot completely rule out the partial 3D contribution of the bulk SOC. But, the WAL induced by 2D surface states depends only on the perpendicular component (θ = 90°) of the applied magnetic field^21^. Sharpness of cusps observed at zero magnetic field depends on the value of *l*_ϕ_, which is the characteristic parameter for quantum interference effects. This length is a measure of the quality of the films as it governs the phase-coherent transport that can be destroyed by inelastic scattering. The variation of magneto-conductivity, Δσ_xx_ = σ_xx_(B) − σ_xx_(B = 0), obtained at 2 K, as a function of the perpendicular component (θ = 90°) of the applied magnetic field (−0.15 T to +0.15 T) is shown in Fig. 2b. In order to get a deeper understanding of WAL phenomena, a more computable analysis is necessary. In a low mobility and strong spin-orbit interaction regime, the quantum correction to the 2D magneto-conduction data is suitably described by HLN theory^22^:

1 $${{\rm{\Delta }}{\rm{\sigma }}}_{{\rm{xx}}}=\frac{\alpha {e}^{2}}{2{\pi }^{2}\hslash }[\mathrm{ln}\,\frac{{B}_{\phi }}{B}-{\rm{\Psi }}(\frac{1}{2}+\frac{{B}_{\phi }}{B})]$$

where, ${\rm{\Psi }}$ is the digamma function, ${B}_{\phi }=\,\frac{\hslash }{4e{l}_{\phi }^{2}}$ and *l*_ϕ_ is the phase coherence length and the other constants like$\,\hslash$, e have their usual meanings. The prefactor α = −1/2^23^^–^^25^ for each transport channel, which has a π berry phase in WAL was obtained after fitting the experimental data to Eq. (1). We get parameters *α* = −0.52, *l*_ϕ_ ~ 470 nm, which confirm the 2D nature of WAL. Such a high value of phase coherence length indicates high quality of the DPB thin film sample. The value of *l*_ϕ_ decreases from 470 nm to 220 nm with increment of temperature from 2 K to 10 K, as was observed in other TI systems^14,15,24^. Prefactor *α* ~ −0.50 is almost independent of temperature in the range of 2 K to 10 K as shown in the inset of Fig. 2c. It indicates the robustness of surface states coupled through bulk which result in a single effective channel for the phase-coherent transport^26,27^. Dimensionality of the system can also be confirmed from the temperature dependence of the *l*_ϕ_. Theoretically for electron-electrons scattering, the coherence length is proportional to the temperature as *l*_ϕ_ ~ T^−1/2^ for the 2D system, *l*_ϕ_ ~ T^−1/3^ for the 1D and *l*_ϕ_ ~ T^−3/4^ for the 3D system^28^. Figure 2c shows temperature dependence power law fit to the *l*_ϕ_ values and it shows *l*_ϕ_ ~ T^−0.46^ which is very close to the expected −0.5 value for the 2D surface states^16^.

### SdH oscillations in DPB

**Figure 3:**
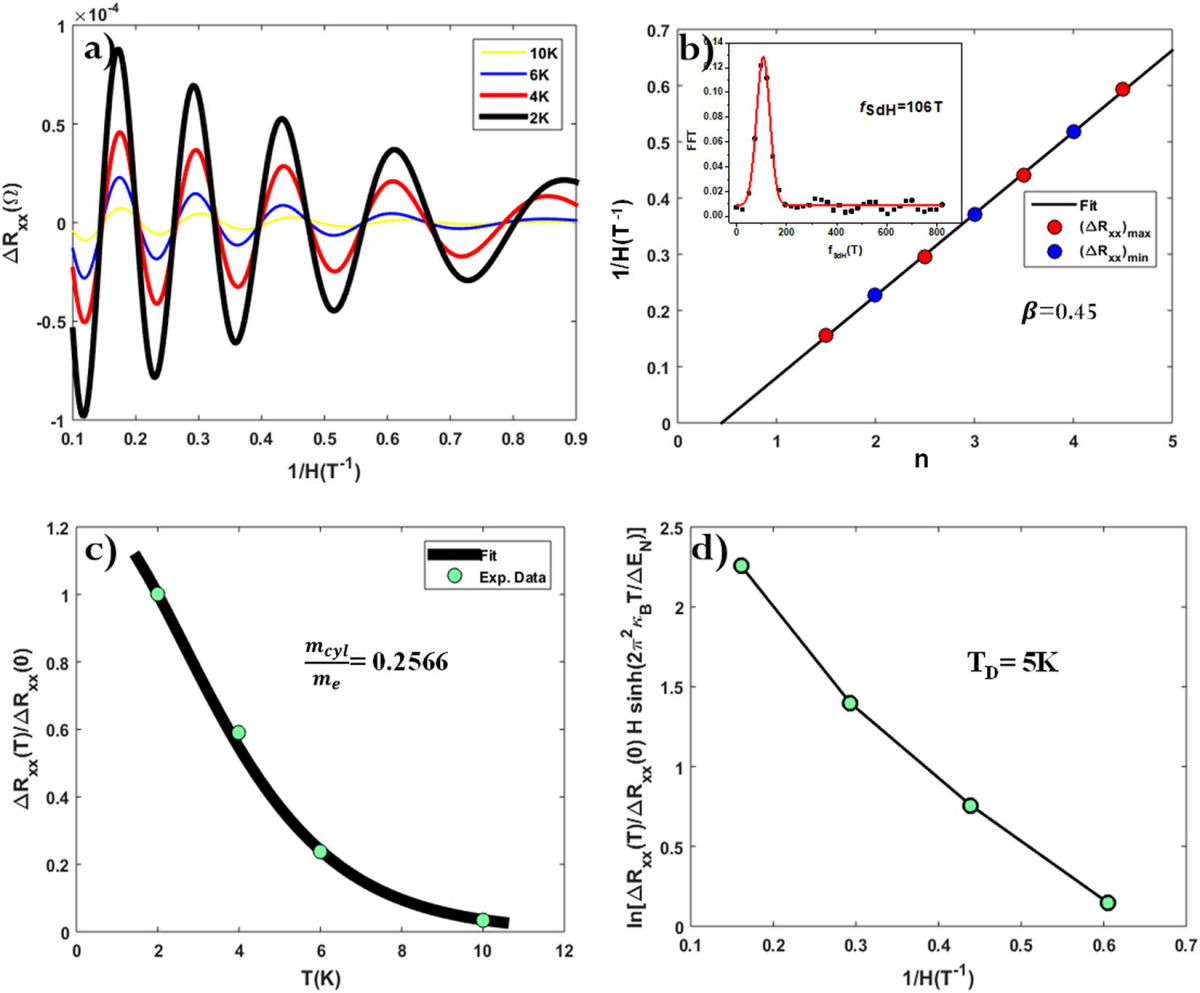
SdH oscillations in DPB. (**a**) SdH oscillations at different temperatures, data fitted after subtracting background and then fitting higher order polynomial equations. (**b**) Landau-level fan diagram for oscillations in ΔR_xx_ measured at 2 K. Least square fitting of the periodic maxima and minima as afunction of the Landau level index (n) gives an intersect at n axisas *β* ~ 0.45 ± 0.1, equivalent to a finite Berry’s phase of 0.90π ± 0.16. Inset of figure shows *f*_SdH_ ~ 106 T obtained after FFT of ΔR_xx_ raw data. (**c**) Experimentally obtained temperature dependent normalized longitudinal resistance as ΔR_xx_(T)/ΔR_xx_(0) (cyan color dots). Fit to the Lifshitz-Kosevich formula at 6.25 T magnetic field (black solid line), yields effective cyclotron mass of carriers as ~0.2566 m_e_. (**d**) Dingle plot of (ΔR_xx_(T)/ΔR_xx_(0)) H sinh(${\lambda }_{D}T/\Delta {E}_{n})$ vs 1/H at 2 K, which gives dingle temperature T_D_ = 5 K.

Quantum oscillations occurring in conductivity such as SdH oscillations are the convincing tools for the characterization of the 2D surface states in TIs^29^. SdH oscillations play particularly important role to quantitatively characterize 2D surface states^17,19,30^ that coexist with 3D bulk states^31^. Additionally, the Berry phase of the system can be extracted from the phase factor of the oscillations which allows us to interpret whether the electrons showing the SdH oscillations are Dirac fermions or not. Hence, we carried out low-temperature; high magnetic field magneto-transport measurements to experimentally verify the surface dominated quantum transport in DPB. The magnetic field was applied at 90° to both the surface of the DPB thin film and the direction of current flow. The variation of MR with magnetic field in Fig. 2a and raw data of longitudinal resistance R_xx_ (Supplementary Fig. S5a) show traces of SdH oscillations, indicating the high carrier mobility at high fields. Smooth background was subtracted from the longitudinal resistance R_xx_ and then the higher order polynomials were fitted to ΔR_xx_ (experimental raw data) (Supplementary Fig. S6a). Figure 3a displays the fitted oscillatory part of R_xx_ (ΔR_xx_) having periodic valleys (minima) and peaks (maxima) as a function of 1/H, indicating high carrier mobility and presence of a well-defined Fermi surface^32,33^. Amplitude of oscillations decreases with decreasing perpendicular magnetic field; also oscillations survive up to 10 K. A single frequency (*f*_*SdH*_ ~ 106 T) was obtained from the Fast Fourier Transform (FFT) analysis of the quantum oscillations in ΔR_xx_(Supplementary Fig. S6). The Onsager relation relates cross section (*A*_F_) of the Fermi surface to *f*_SdH_ in momentum space as $\,{f}_{SdH}=(\frac{h}{4{\pi }^{2}e}){A}_{F}$, where *A*_*F*_ = π${k}_{F}^{2}$, *h* is the Planck’s constant; *k*_F_ is the Fermi vector and *e* is the electron charge. For a *2D* Fermi surface system, *k*_F_ is related to the sheet carrier concentration (n_s_) by relation (${n}_{s}=\frac{{k}_{F}^{2}}{4\pi })$. Sheet carrier concentration (*n*_*s*_ ~ 2.56 × 10^12^ cm^−2^) was calculated from the Fermi vector *k*_F_ ~ 0.0568 Å^−1^, corresponding to *f*_SdH_ ~ 106 T. This calculated carrier concentration (*n*_*s*_ ~ 2.56 × 10^12^ cm^−2^) from SdH oscillations (for assumed *2D* cross section *A*_*F*_) is consistent with the experimentally obtained carrier concentration from Hall measurements (*n* ~ 2.71 × 10^12^ cm^−2^ discussed later). Hence, we may conclude that these SdH oscillations are originating from the *2D* surface states.

In SdH oscillations, longitudinal conductivity oscillates periodically as a function of 1/H and follows the relation:

2 $${\sigma }_{{\rm{xx}}} \sim cos[2{\rm{\pi }}(\frac{{f}_{SdH}}{{H}_{n}}-\frac{1}{2}+\beta )]$$

where, H_n_ is the magnetic field at *n*^th^ landau level, ${f}_{SdH}$ is the SdH oscillation frequency and *β* is simply the Berry Phase (*γ*) divided by 2π. Dirac fermions possessing a linear energy dispersion^26,34^ have Berry’s phase ~π with *β* = 1/2. The phase factor *β* can be calculated experimentally from SdH oscillations using the so called Landau Level (LL) Fan diagram^35–37 ^analysis. In our analysis we plotted 1/*H*_*n*_ values corresponding to the maxima and minima in amplitude of Δ*R*_*xx*_ vs landau level index (*n*) assigned to them^35^, as shown in Fig. 3b. From Eq. 2, linear fit to LL Fan diagram of 1/*H*_*n*_ vs *n* for a straight line, slope of this line gives ${f}_{SdH}$ and intercept on *n*-index axis gives *β*. To determine the Berry phase from the LL fan diagram^23,38^, we fix the slope of the linear fitting by using the frequency ${f}_{SdH}$ ~ 106 T obtained from the Fourier analysis of the data at 2 K (shown in Fig. 3b) and obtain β = 0.45 ± 0.1, corresponding to Berry phase 0.90π ± 0.16. In this study the error of ±0.1 in *β* and ±0.16 in Berry’s phase is a conventional estimate of the error in determining the positions of maxima and minima in the data shown in the Fig. 3a and is relatively small as the slope is fixed in the analysis.

Temperature dependent amplitude of SdH oscillations in *ΔR*_*xx*_, can be further fitted to the standard Lifshitz-Kosevich theory^26^, expressed as:

3 $${\Delta }{R}_{xx}(T,H)\propto exp(\frac{-{\lambda }_{D}{T}_{D}}{\Delta {E}_{n}(B)})\times \frac{{\lambda }_{D}T/\Delta {E}_{n}(B)}{\sinh ({\lambda }_{D}T/\Delta {E}_{n}(B)}$$

Here ${T}_{D}$ and $\Delta {E}_{n}(B)$ are the fitting parameters, ${\lambda }_{D}=2{\pi }^{2}$κ_B_, where κ_B_ is the Boltzmann constant and H is the magnetic field coordinate for the *n*^*th*^ maximum in *ΔR*_*xx*_. The energy gap between n^th^ and (n + 1)^th^ Landau level, is given by *ΔE*_*n*_(*B*) = $\hslash \,$e*H*/*m*_*cycl*_ with m_*cycl*_ as cyclotron mass of carriers (electron), *h* Planck’s constant and e is electron charge. *T*_*D*_ = ћ/2π*τ*κ_B_ is dingle temperature and *τ* is the transport lifetime of the surface states. We perform best fit as shown in Fig. 3c to ΔR_xx_(T)/ΔR_xx_(0) experimental data (Cyan dots) from Eq. (3) (Black solid curve). Extracted parameters are obtained as$\,\Delta {E}_{n}(B)$ = 2.8 meV which further gives m_*cycl*_ = 0.2566 m_e_ (m_e_ is free electron mass ~9.1 × 10^−31^ kg). Fermi level(E_F_) is related to m_*cycl*_ as *E*_*F*_ = *m*_*cycl*_*V*_*F*_^2^, where, *V*_*F*_ is the Fermi velocity and is related to Fermi vector (*k*_*F*_) as *V*_*F*_ = $\hslash \,$*k*_*F*_/*m*_*cycl*_^33,39^. This equation gives *V*_*F*_ ~ 2.5621 × 10^5^ ms^−1^ indicating that the Fermi level (*E*_*F*_) lies at (*E*_*F*_^S^ ~ 96 meV) above the Dirac point. Dingle plot fitting reveals more accurate estimation of transport lifetime(*τ*) of the surface states^35,40^. Slope of the semi-log plot of *(ΔR*_*xx*_*(T)/ΔR*_*xx*_*(0)) H sinh(*${\lambda }_{D}T\,/\,\Delta {E}_{n})\,$vs *1/H* gives dingle temperature *T*_*D*_. As shown in Fig. 3d best fit of Dingle Plot gives *T*_*D*_ ~ 5 K, and carrier lifetime *τ* ~ 2.5963 × 10^−13^ s. Further, mean free path of carriers *l* ~ 67 nm (*l* = *V*_*F*_*τ*) and mobility *µ*_*s*_ ~ 1780 cm^2^V^−1^s^*−*1^ (*µ*_*s*_ = *eτ/m*_*cycl*_) was estimated. Such a high mobility of carriers and small cyclotron mass of electron suggest dominant surface transport.

### Temperature-dependent longitudinal and hall resistivity in DPB

**Figure 4:**
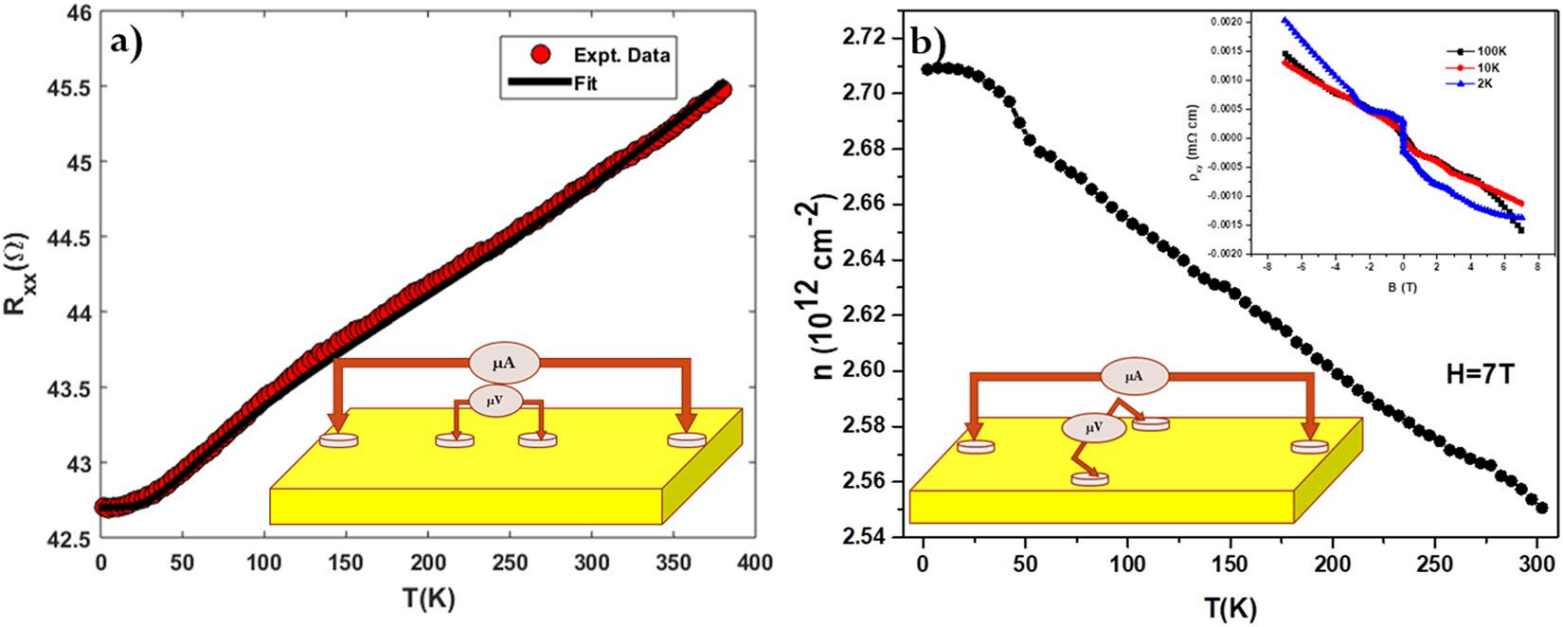
Electrical transport properties of DPB. (**a**) Temperature-dependent longitudinal resistance (R_xx_) measurement in zero magnetic field (red data points), black line is fitting of simple resistivity Eq. ${R}_{xx}=\,{R}_{o}+A{T}^{5}+\,B\,\exp (\,-\,\frac{\theta }{T})+\gamma {T}^{2}$. (**b**) Variation of carrier concentration (deduced from Hall effect measurement) with temperature. The inset shows the Hall resistivity (*ρ*_xy_) of DPB as a function of magnetic field at temperatures 2 K, 10 K and 100 K. The dominant charge carriers are electrons.

In Fig. 4a, we show the temperature dependence of the zero-field raw data of longitudinal resistance R_xx_, in the temperature range 2 K ≤T≤ 380 K. Above 25 K a metallic behavior with a very small change in temperature coefficient of resistivity, characteristic of a degenerately doped semiconductor, is observed. Dependence of *R*_*xx*_ on *T* can be well fitted using a resistivity equation^41,42^ given as:

4 $${R}_{xx}=\,{R}_{o}+A{T}^{5}+B\,\exp (-\frac{\theta }{T})+\gamma {T}^{2}$$

where, *R*_*o*_ corresponds to a low temperature residual resistance that arises due to impurities/imperfections scattering. The term AT^5^ in Eq. 4 represents the normal e-phonon (*ph*) scattering below the Debye temperature^42^. Now if phonons drift along with electrons, the *ph*-drag contribution becomes significant and coefficient A reduces considerably. The exponential term in the equation describes the e-*ph* umklapp scattering and is unaffected by *ph*-drag^42–44^. The quadratic term (T^2^) in Eq. 4 is the result of electron–electron (*e-e*) scattering^45,46^. The fit of *R*_*xx*_ − *T* data in Eq. 4 yields the fitting parameters as *R*_*o*_ = 42.7 Ω, A = 10^−16^, *B* = 1.7 Ω, *θ* = 95 K and *γ* = 0.0000107 Ω/K^2^, corresponding to the best fit (shown using a black color solid line) to the experimental raw data shown in Fig. 4a. Since there is very small change in resistance with increase in temperature, coefficient A approaches to zero which indicates considerable *ph*-drag effects, hence large angle e-*ph* scattering parameter T^5^ is absent here^47,48^. Next, the extremely small value of *γ* indicates very less *e*-*e* scattering in the sample and hence the transport is mainly dominated by e-*ph* scattering^49^. Figure 4b shows the temperature dependence of carrier concentration derived from the Hall Effect measurements in hall probe geometry (shown in the lower inset of Fig. 4b).

Sheet carrier concentration extracted from the Hall data at 2 K, *n* ~ 2.71 × 10^12^ cm^−2^, matches well with the data obtained from SdH oscillations in the magneto-transport data. Carrier concentration remains relatively flat till temperature 25 K and starts decreasing above 25 K. The change in career concentration for a temperature between 2 K and 300 K is very small ~5.8%. The upper inset of Fig. 4b shows the Hall resistivity *ρ*_*xy*_ of DPB as a function of magnetic field at temperatures 2 K, 10 K and 100 K. Negative slope observed in *ρ*_xy_ indicates that the dominant charge carriers are electrons.

## Discussion

In summary, a thorough study of the magneto-transport properties of 20 nm half-Heusler DPB thin film reveals the presence of 2D edge states.

The resistance *versus* temperature plot shown in Fig. 4 clearly shows that although at higher temperatures (above 25 K), metallic behavior is observed in our thin-film; a relatively flat variation in *R*_*xx*_ with temperature is seen up to T ~ 25 K. Such saturation-like behavior in resistance at low-temperatures, is a commonly observed phenomenon in topological semimetals^46,48–50^. It should be noted that for DPB polycrystalline bulk sample, an anamoly at around 3 K, in resistivity versus temperature curve (during cooling below 3 K) has been reported in literature^11,51^. The enhanced resistivity below 3 K observed in the reported bulk sample has been attributed to the antiferromagnetic transition observed at around the same temperature (T_N_ ~ 3.5 K) in them^51^. In our thin film DPB samples, no discernible anomaly was observed at low temperatures (below 25 K); possibly due to the surface conduction dominated transport in our thin-film sample.

**Figure 5:**
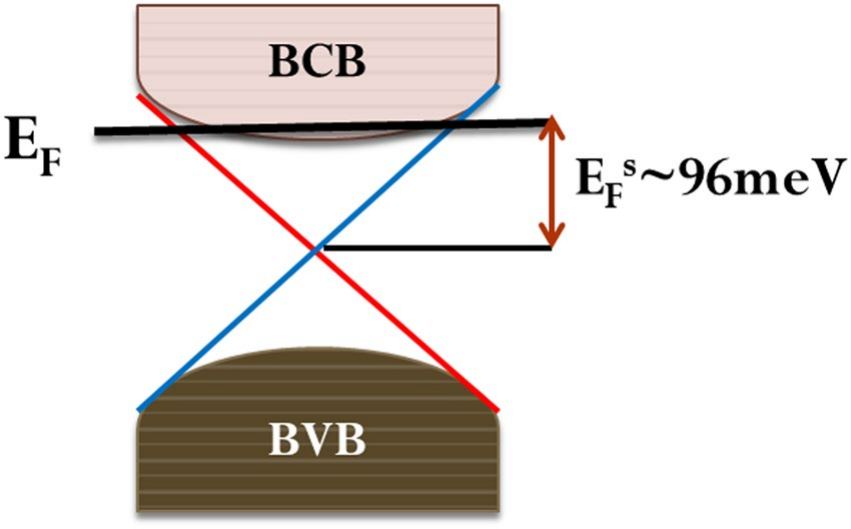
Band diagram of DPB thin film. Schematic picture of the bulk and surface band structures, here chemical potential (the thick black horizontal line) lies in the bulk conduction band. The Fermi level E_F_ lies at ~96 meV above the Dirac point. BCB- Bulk conduction band, BVB- Bulk valence band.

Fitting parameters obtained from the resistance *Vs* temperature data indicate that below 25 K *e-e* scattering dominates the resistivity behavior. This is possibly arising from either the presence of disorder on the surface of TI leading to *e-e* correlation among surface states or due to freezing of bulk carriers in the impurity bands^52^. Above 25 K, evidently the resistivity behavior can be explained by *e-ph* scattering and *ph*-drag effects govern the resistivity behavior. This also explains the metallic behavior of DPB thin films at higher temperatures that differ significantly from bulk sample resistivity behavior^11,15^. Hall measurements reveal the dominance of *n* type carriers (electrons). The 2D WAL effect observed near magnetic field B = 0 in the magneto-conductivity data is a key signature of TIs. The magneto-transport data at 2 K (shown in Fig. 2b) fitted very well with HLN theory near B = 0. A reasonably large value of phase coherence length (*l*_ϕ_ ~ 470 nm) indicates a very small *e-e* scattering and is consistent with the observation of small *γ* values obtained from resistivity data. The value of *l*_ϕ_ decreases monotonically with increase in temperature as *l*_ϕ_ ~ T^−0.46^, which is very close to theoretically predicted *l*_ϕ_ ~ T^−0.50^ dependence for 2D TI^24^. This power law dependence of *l*_ϕ_ is a manifestation of dephasing of phase-coherent transport of carriers by phonons on the surface of the film. From HLN theory fitting of magneto-transport data we obtained the value of the prefactor α ~ −0.52, which is almost equal to the theoretical value of α ~ −0.50 expected for the WAL in 2D systems^53^. Clear SdH oscillations with single frequency (*f*_*SdH*_ ~ 106 T) were also observed in the low temperature and high magnetic field MR data. The calculated value of the carrier density (*n*_*s*_ ~ 2.56 × 10^12^ cm^−2^) from SdH oscillations are consistent with the carrier concentration (*n* ~ 2.71 × 10^12^ cm^−2^) obtained from Hall measurement. After fitting of the magneto-transport data using standard Lifshitz-Kosevich theory, various parameters were extracted as shown in Table 1, and these results allow us to draw schematics of the band diagram of DPB as shown in Fig. 5.

**Table 1:** Parameters obtained from the SdH oscillations at T = 2 K.

*f*_SdH_ (T)	N_2D_ (10^12^ cm^−2^)	m_cycl_ (m_e_)	k_F_ (Ȧ^−1^)	V_F_ (10^5^ ms^−1^)	E_F_^s^(meV)	τ (10^−13^ s)	*l* (nm)	µ_s_ (cm^2^V^−1^s^−1^)
106	2.56	0.2566	0.0568	2.5621	~96	2.5963	~67	~1780

From estimated parameters in Table 1, considerably small value of free electron mass (0.2566 m_e_) and a reasonably high mobility (~1780 cm^2^V^−1^s^−1^) were obtained for the 3 mm × 5 mm × 20 nm DPB film. These values are similar to the earlier reports on single crystal TIs^17,19,20^ as well as thin film TI samples^7,23,33^. As stated by Ando *et al*.^53,54^, if surface carriers have high enough mobility, surface dominated transport in the sample is indicated. The 2D nature of WAL in our DPB film was also confirmed by power law dependence of phase coherence length^16^, *l*_ϕ_ ~ T^−0.46^. Also, as shown above in the Results section, the carriers in the DPB film carry a Berry phase of ~0.90π; all the above facts imply that the observations of SdH oscillations and WAL effect in magneto-transport data are due to the presence of Dirac fermions^35,39,55^. Metallic behavior of the sample indicates that Fermi level (E_F_) lies within the bulk conduction band (BCB) and from SdH oscillations we find out that it lies at (*E*_*F*_^S^ ~ 96 meV) above the Dirac point (formed by edge states), see Fig. 5.

Thin film formation leads to (~0.2%) tensile strain in the film, due to a lattice mismatch of DPB (110) with MgO(100)substrate. Also, thin film deposition in vacuum and oriented film formation itself leads to additional strain^56,57^. The increase in lattice constant of DPB due to strain reduces the hybridization and closes the non-zero band gap; combined with the high SOC due to Bi, this possibly leads to the transition from trivial to TI state. As Chadov *et al*.^10^ have shown for YPdBi, increase in lattice constant by ~0.3% leads to the inverted state i.e. TI state. More detailed study is yet required for exact quantification of strain values that are required to drive these Half Heusler alloys in to TI state located at the border line between trivial and TI states.

## Methods

### Sample preparation

Thin films of DPB were grown in a two-step process. The first step was to prepare polycrystalline samples of stoichiometric DPB by specially designed RF induction melting method, using optimized amounts of the constituent elements in a high-purity argon atmosphere. A cooled water flow was maintained in the copper base holder to keep the alloy cool. Dy (99.95%) pieces, Pd (99.95%) granules, and Bi (99.999%) chunks were used as starting elements. Second step was DPB thin film preparation using pulsed laser deposition (PLD) technique. The polycrystalline powder sample was pelletized in 1” PLD target. Base pressure of 2 × 10^−6^ Torr was maintained in the PLD chamber. MgO(100) substrate (of dimension ~3 mm × 5 mm) with Ta (5 nm) as seed layer was used for sample preparation. Various PLD parameters like, laser plume energy, substrate temperature (500 °C), target to substrate distance, frequency and number of laser shots were optimized to get best samples of required composition and thickness.

### Materials characterization

The crystal structure, surface morphology, composition and film thickness of the grown thin films were investigated using X-ray diffraction, AFM, EDX and X-Ray reflectivity (XRR) respectively. XRD, XRR and rocking curve measurements were performed using Cu K_α_ PANalytical X’Pert Highscore diffractometer. XRD analysis showed a textured structure with the (110) orientation. Film morphology, topographical scanning was performed using the Nanoscope IIIa, M/S Digital Instruments (Dimension 3100) equipped with a phase extender box. ZEISS EVO Series SEM (EVO 50) which has RONTEC’s EDX system Model (QuanTax 200) and provides an energy resolution of 127 eV at Mn K_α_was used for the EDX analysis.

### Transport measurements

Transport measurements were performed on rectangular samples with dimensions 3 mm × 5 mm × 20 nm; ohmic contacts were made with silver paste and cured at room-temperature. Longitudinal resistivity (*ρ*_*xx*_) and Magneto-resistance data of thin film sample were measured using the four-probe method with a dc-gauge current of 500 µA in a commercial Physical Properties Measurement System (PPMS-7T) from Quantum Design which can sweep the magnetic field between ± 7T at lowest temperature up to 2 K. Hall resistance data were measured using Multiutility probe (MUP) modified for electrical measurements in SQUID MPMS XL7 from Quantum design.

## Electronic supplementary material

Supplementary information
