# Supplementary material for: Weak Antilocalization and Quantum Oscillations of Surface States in Topologically Nontrivial DyPdBi(110)Half Heusler alloy
Source: Sci Rep. 2018 Jul 2;8:9931. doi: 10.1038/s41598-018-28382-1 (PMC6028386; doi:10.1038/s41598-018-28382-1)
Supplement: Supplementary file 1 — Supplementary information [file 41598_2018_28382_MOESM1_ESM.docx]

**Weak Antilocalization and Quantum**

**Oscillations of Surface States in Topologically Nontrivial**

**DyPdBi(110) Half Heusler alloy**

Vishal Bhardwaj^1^, Satyendra Prakash Pal^1^, Lajos K. Varga^2^, Monika Tomar^3^, Vinay Gupta^3^, Ratnamala Chatterjee^1*^,

^1^Department of Physics, Indian Institute of Technology Delhi, HauzKhas, New Delhi-110016, India

^2^Wigner research center for Physics Hungarian Academy of Sciences, P.O.B. 49, H-1525 Budapest, Hungary

^3^Department of Physics and Astrophysics, University Of Delhi, Delhi, 110007, India

[^*^ratnamalac@gmail.com](mailto:*ratnamalac@gmail.com), rmala@physics.iitd.ac.in

**Supplementary Information**

1. **Figure S1| (a) X-Ray diffraction of DyPdBi thin film at different substrate temperatures.(b)An architecture showing growth of <110> DyPdBi atoms on top of MgO(100) atoms.**
2. **Figure S2|** **X Ray Reflectivity spectra of thin films of thickness (sample S1~15nm and S2~20nm) was estimated.**
3. **Figure S3|** **A scanning electron microscope with energy-dispersive X-ray spectrometry (SEM-EDX) for sample S1.**
4. **Table S1|** **Elemental composition of DyPdBi thin film obtained by EDX for sample S1.**
5. **Figure S4|** **A scanning electron microscope with energy-dispersive X-ray spectrometry (SEM-EDX) for sample S2.**
6. **Table S2|** **Elemental composition of DyPdBi thin film obtained by EDX for sample S2.**
7. **Figure S5| (a) Observation of SdH oscillations in raw longitudinal resistance (R_xx_) data for sample S2. (b)** **Normalized MR as a function of magnetic field H at a series of temperatures T =2, 4, 6 and 10 K for sample S1.**
8. **Figure S6|Higher order polynomial fitting in linear background subtracted magneto resistance data (ΔR_xx_) for sample S2. (b) Fast Fourier transform (FFT) spectra of SdH oscillations for sample S2.**

**Figure S1| (a) X-Ray diffraction of DyPdBi thin film at different substrate temperatures, for sample S2 with thickness 20nm.(b)An architecture showing growth of <110> DyPdBi atoms on top of MgO(100) atoms.**

**Figure S2|** **X Ray Reflectivity spectra of DPB thin films (a) Sample S1 with thickness ~15nm. (b) Sample S2 with thickness ~20nm (used in manuscript for analysis).**

**Figure S3|** **(a)** **A scanning electron microscope image of selected spectrum:** Object 3241**, sample S1. (b) Energy-dispersive X-ray spectrometry for sample S1 corresponding to selected region, as shown in table S1.** The composition is fairly uniform in different regions of thin film for sample S1.

**Table S1|** **Elemental composition of DyPdBi thin film obtained by EDX for sample S1.**

| El | AN | Series | Atom. C | Error |
| --- | --- | --- | --- | --- |
|  |  |  | [ at. % ] | [%] |
| Mg | 12 | K-series | 39.01 | 2.8 |
| O | 8 | K-series | 55.27 | 13.1 |
| Bi | 83 | L-series | 1.32 | 1 |
| Ta | 73 | L-series | 1.79 | 2.1 |
| Dy | 66 | L-series | 1.30 | 1.2 |
| Pd | 46 | L-series | 1.31 | 1.1 |
|  |  | Total: | 100 |  |

Calculated average atomic % of Dy, Pd, Bi is **32.66%,** **33.33% and 34.01%,** respectively for different regions of sample S1.

**Figure S4|** **(a)** **A scanning electron microscope image of selected spectrum:** Object 3243**, sample S2. (b) Energy-dispersive X-ray spectrometry for sample S2 corresponding to selected region, as shown in table S2.** The composition is fairly uniform in different regions of thin film for sample S2.

**Table S2|** **Elemental composition of DyPdBi thin film obtained by EDX for sample S2.**

| El | AN | Series | Atom. C | Error |
| --- | --- | --- | --- | --- |
|  |  |  | [ at. % ] | [%] |
| Mg | 12 | K-series | 35.05 | 2.8 |
| O | 8 | K-series | 55.27 | 13.1 |
| Bi | 83 | L-series | 2.85 | 1 |
| Ta | 73 | L-series | 1.18 | 2.1 |
| Dy | 66 | L-series | 2.81 | 1.2 |
| Pd | 46 | L-series | 2.84 | 1.31 |
|  |  | Total: | 100 |  |

Calculated average atomic % of Dy, Pd, Bi is **33.08%,** **33.42% and 33.50%,** respectively for different regions of sample S2.

**Figure S5| (a) Observation of SdH oscillations in raw longitudinal resistance (R_xx_) data for sample S2. (b)** **Normalized MR as a function of magnetic field H at a series of temperatures T =2, 4, 6 and 10 K for sample S1.**

**S2**

**S2**

**Figure S6|Higher order polynomial fitting in linear background subtracted magneto-resistance data (ΔR_xx_) for sample S2. (b) Fast Fouier transform (FFT) spectra of SdH oscillations with single frequency *f*_SdH_ ~106T for different temperatures (2K, 4K, 6K and 10K) for sample S2.**
